# Pyk2 in the amygdala modulates chronic stress sequelae via PSD-95-related micro-structural changes

**DOI:** 10.1038/s41398-018-0352-y

**Published:** 2019-01-15

**Authors:** Enrica Montalban, Omar Al-Massadi, Anna Sancho-Balsells, Verónica Brito, Benoit de Pins, Jordi Alberch, Silvia Ginés, Jean-Antoine Girault, Albert Giralt

**Affiliations:** 1 Inserm UMR-S 839, 75005 Paris, France; 2 Sorbonne Université, Faculté des Sciences et d’Ingénierie, 75005 Paris, France; 3 Institut du Fer a Moulin, 75005 Paris, France; 4 Department of Physiology, CIMUS, University of Santiago de Compostela-Instituto de Investigación Sanitaria, 15782 Santiago de Compostela, Spain; 5 CIBER Fisiopatología de la Obesidad y Nutrición (CIBERobn), 15706 Santiago de Compostela, Spain; 6 Departament de Biomedicina, Facultat de Medicina, Institut de Neurociencies, Universitat de Barcelona, 08036 Barcelona, Spain; 7 Institut d’Investigacions Biomèdiques August Pi i Sunyer (IDIBAPS), 08036 Barcelona, Spain; 8 Centro de Investigacion Biomedica en Red Sobre Enfermedades Neurodegenerativas (CIBERNED), 28031 Madrid, Spain

## Abstract

Major depressive disorder (MDD) is a common disorder with a variety of symptoms including mood alterations, anhedonia, sleep and appetite disorders, and cognitive disturbances. Stressful life events are among the strongest risk factors for developing MDD. At the cellular level, chronic stress results in the modification of dendritic spine morphology and density. Here, we study the role of Pyk2 in the development of depressive-like symptoms induced by a model of chronic unpredictable mild stress (CUMS). Pyk2 is a non-receptor calcium-dependent protein-tyrosine kinase highly expressed in the forebrain principal neurons and involved in spine structure and density regulation. We show that Pyk2 knockout mice are less affected to anxiety-like and anhedonia-like phenotypes induced by the CUMS paradigm. Using region-specific knockout, we demonstrate that this phenotype is fully recapitulated by selective Pyk2 inactivation in the amygdala. We also show that in the absence of Pyk2 the spine alterations, PSD-95 clustering, and NMDA receptors changes induced by the CUMS paradigm are prevented. Our results reveal a possible role for Pyk2 in the response to stress and in synaptic markers expression and spine density regulation in the amygdala. We suggest that Pyk2 contributes to stress-induced responses through micro-structural changes and that its deficit may contribute to the resilience to chronic stress.

## Introduction

Major depressive disorder (MDD) is a debilitating disease characterized by low mood, loss of interest in outside stimuli, impaired cognitive function, and vegetative symptoms, such as disturbed sleep or appetite^1^. MDD is a multifactorial and clinically heterogenous mental disorder with a percentage of heritability estimated around 35% and yearly incidence of 6% of affected individuals among the adult worldwide population^2^. Chronic life stressors are among the main risk factors for developing a major depressive syndrome^3^. Notably, a clear association between stressors and the emergence of major depressive episodes has been demonstrated among dysthymic patients^4^. The impact of stressors depends on the characteristics of the stressor itself (e.g. severity, chronicity, predictability), coping ability, and individuals’ stressor history (including early life trauma)^3,5–7^.

A large body of evidence shows that in animal models depression is closely associated to microstructural alterations in dendritic spines morphology and spine density^8^. Indeed, chronic stress results in decreased dendritic spine density in hippocampus and prefrontal cortex, and an increased spine density in amygdala and nucleus accumbens^9–12^. Therefore, understanding dendritic spines dynamics and turnover and identifying master molecules regulating this phenomenon is crucial for uncovering the mechanisms underlying stress-induced depression.

Pyk2 could be one of these master molecules. Pyk2 is a non-receptor tyrosine kinase that can be activated by Ca^2+^ and is highly expressed in cortex, amygdala, striatum, and hippocampus^13,14^. Previous findings indicated a role for Pyk2 in the regulation of hippocampal synaptic plasticity^15–17^. Our recent work with Pyk2 mutant mice showed its importance in vivo for spatial learning and memory, synaptic plasticity and spine density, and morphology^18^. Yet, the role of Pyk2 in the physiopathology of MDD remains largely unexplored as only two reports have pointed out a possible role of Pyk2 in this context. First, it has been reported that a model chronic stress in mice increases the colocalization of Pyk2 with NUP62, a nucleoprotein involved in chromatin organization and transcription^19^. The increased colocalization results in an increased phosphorylation of NUP62^19^ suggesting a potential role of Pyk2 in mediating depressive-like symptomatology after chronic stress via the indirect regulation of gene transcription. A second study demonstrated that, in a model of chronic stress in rats, the over-expression of Pyk2 in the lateral septal nucleus has a positive effect on the forced swimming test’s performance^20^, a common paradigm to evaluate behavioral despair in rodents, one of the symptoms of MDD^21^. Finally, a recent genome wide association study (GWAS) indicated that the *PTK2B* gene is significantly associated with neuroticism^22^ which in turn is a common risk factor to develop MDD^23^. Despite these early results, the relevance of the tyrosine kinase Pyk2 in the pathophysiology of MDD is not known. Understanding in which brain region and through which molecular mechanism Pyk2 regulates depressive symptoms would provide important insights on the possible role of PyK2 in MDD.

We recently generated full knockout mice for the *PTKB2* gene, as well as mice with floxed *PTK2B* alleles, which permit to inactivate the gene in discrete brain regions or cell types^13,18^. Mice were exposed to the chronic unpredictable mild stress (CUMS) paradigm to induce depressive-like symptoms. The CUMS protocol overcomes the stress habituation and has been widely used to model depression-like behaviors^8,24^. As expected, we observed that CUMS induced a severe depressive-like performances in the stressed mice as compared to non-stressed controls. Observed phenotypes included; decreased time spent in the open arms in the plus maze, decreased time struggling in the forced swimming test, and decreased preference for sweet water in the sucrose preference test as described previously^25^. Surprisingly, compared to WT mice, Pyk2-deficient mice showed reduced behavioral sequelae induced by the CUMS paradigm in several tests. Region-specific KO studies showed a major role of Pyk2 in the basolateral nuclei of the amygdala. We also examined spine density in this brain region and found that stress-induced spine alterations were absent in Pyk2-deficient mice. These results underline the possible role of Pyk2 in depressive states induced by stress.

## Materials and methods

Detailed descriptions and procedures for genetically modified mice, CUMS paradigm, open field test, elevated plus maze test, forced swimming test, sucrose preference test, viral constructs and stereotaxic injection, tissue preparation and immunofluorescence, confocal imaging and analysis, Golgi staining and spines analysis, immunoblot analysis and statistical analysis are provided in the Supplementary Methods section.

## Results

### Effects of CUMS paradigm in mice lacking *PTK2B* gene (Pyk2^−/−^ mice)

**Fig. 1:**
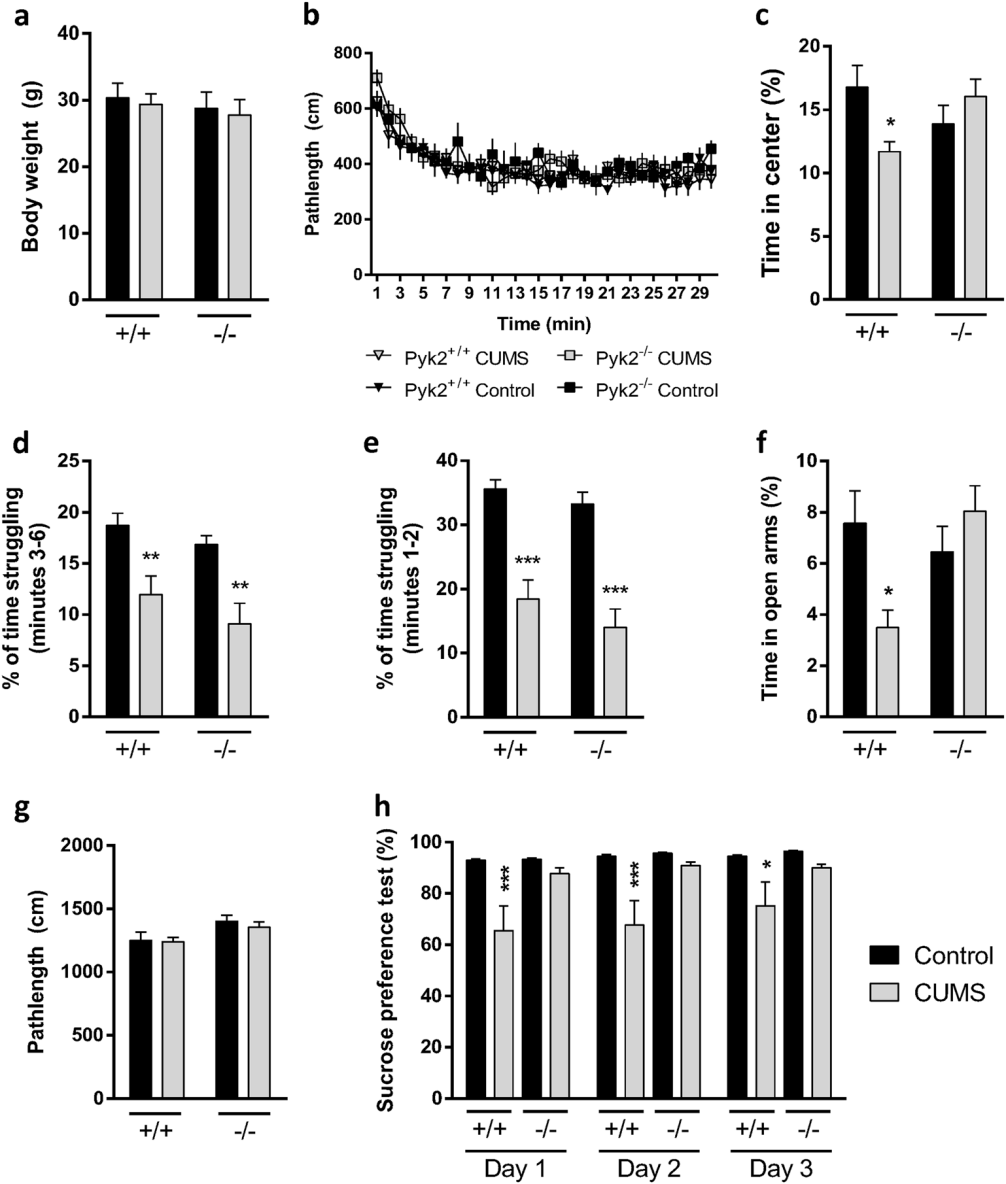
Behavioral effects of CUMS in Pyk2^+/+^ and Pyk2^−/−^ mice. **a** Body weight was monitored as a measure of general health. Two-way ANOVA, genotype effect: *F*_(1, 51)_ = 2.19, *p* = 0.09. **b** Pathlength traveled in an open field apparatus during 30 min. Two-way ANOVA, genotype effect: *F*_(3, 52)_ = 0.303, *p* = 0.82. **c** Time in the center in an open field apparatus during 30 min. Two-way ANOVA, interaction effect: *F*_(1, 52)_ = 6.956, *p* = 0.011. **d** Forced swimming test: % of time struggling from the 3rd to the 6th minute in a 6-min long session. Two-way ANOVA, genotype effect: *F*_(1, 52)_ = 21.72, *p* < 0.0001. **e** Forced swimming test: % of time struggling from the 1st to the 2nd minute in a 6-min long session. Two-way ANOVA, genotype effect: *F*_(1, 52)_ = 54.33, *p* < 0.0001. **f** Time spent in the open arms of an elevated plus maze. Two-way ANOVA, genotype effect: *F*_(1, 52)_ = 4.14, *p* = 0.01. **g** Path length traveled in the elevated plus maze. Two-way ANOVA, genotype effect: *F*_(1, 52)_ = 2.24, *p* = 0.078. **h** Sucrose preference test. Preference for sweet water over plain water (%). Three consecutive days of testing were evaluated in the sucrose preference test. Two-way ANOVA genotype effect: day 1, *F*_(1, 52)_ = 11.20, *p* = 0.0015; day 2, *F*_(1, 52)_ = 10.73, *p* = 0.0019; day 3, *F*_(1, 52)_ = 7.42, *p* = 0.008. Data are means + SEM. Control Pyk2^+/+^ (*n* = 14); control Pyk2^−/−^ (*n* = 14); Pyk2^+/+^:CUMS (*n* = 13) and Pyk2^−/−^:CUMS (*n* = 15). Tukey’s post hoc test was used in all experiments. **c–h**: *p < 0.05, **p < 0.01, and ***p < 0.001 compared to Pyk2^+/+^ controls. CUMS Chronic unpredictable mild stress group

Pyk2^+/+^ and Pyk2^−/−^ mice were exposed for 28 days to randomized and unpredictable aversive stimuli (CUMS protocol, see Materials and methods and supplementary figure 1a). After the CUMS protocol all mice were evaluated for behavioral alterations. Naive mice from both genotypes (Pyk2^+/+^ and Pyk2^−/−^) were used as controls. First, as a general health index, we also monitored the body weight and we observed no difference in any group as compared to Pyk2^+/+^ controls (Fig. 1a). Next, we used the open field to evaluate time spent in the center of the arena and pathlengths in Pyk2^+/+^ and Pyk2^−/−^ mice exposed (Pyk2^+/+^:CUMS and Pyk2^−/−^:CUMS) or not to CUMS (control Pyk2^+/+^ and Pyk2^−/−^ mice). All groups of mice habituated well and showed no difference in pathlengths (Fig. 1b) during the 30-min session. However, Pyk2^+/+^:CUMS but not Pyk2^-/-^:CUMS mice displayed significantly reduced time in the center in the open field paradigm (Fig. 1c). We then subjected the mice to the forced swimming test (FST). We measured the time during which mice were mobile to escape from the water: stressed mice showed lower levels of time struggling as compared to their respective controls without CUMS. No difference was observed when comparing Pyk2^+/+^ and Pyk2^−/−^ control mice or Pyk2^+/+^:CUMS and Pyk2^−/−^:CUMS mice (Fig. 1d). We also analyzed the two first minutes of the assay and no changes were observed when comparing Pyk2^+/+^ and Pyk2^−/−^ control mice or Pyk2^+/+^:CUMS and Pyk2^−/−^:CUMS mice (Fig. 1e). Next, we used an elevated plus maze to evaluate the effect of the CUMS paradigm on time spent in the open arms in the four groups of mice (Fig. 1f, g). We found a significant preference for the open arms of the Pyk2^+/+^ control mice as compared to the Pyk2^+/+^:CUMS (Fig. 1f). Interestingly this difference was lost when comparing control Pyk2^−/−^ mice to Pyk2^−/−^:CUMS, which all spent a comparable amount of time as the Pyk2^+/+^ controls in exploring the open arm. Importantly the locomotor activity (measured as a pathlength) tested in the plus maze was similar in all groups (Fig. 1g). Finally, we evaluated the effect of the genotype on potential CUMS-induced changes in the sucrose preference test. Animals were tested for 3 consecutive days for their preference for sweet water (2% sucrose) versus plain water (Fig. 1h). Control Pyk2^+/+^ mice displayed high levels of preference towards sweet water as compared to normal water. In contrast, although they still had some preference for sweet water, Pyk2^+/+^:CUMS mice displayed a significantly reduced preference as compared to Pyk2^+/+^ unstressed controls. Intriguingly, Pyk2^−/−^ and Pyk2^−/−^:CUMS groups of mice showed a comparable preference for sweet water, with no difference from that observed in control Pyk2^+/+^ mice. This set of results indicates that behavioral alterations induced by the CUMS paradigm in Pyk2^+/+^ mice were almost completely absent in Pyk2^−/−^ mice in the same conditions. In contrast, the performance in the forced swimming test was not affected.

### Pyk2 inactivation in the amygdala is sufficient to mimic the Pyk2^−/−^ mice phenotype after CUMS

To gain insight on the brain region that could mediate such behavioral changes we knocked out Pyk2 in the neurons of selected brain regions by injecting Pyk2^f/f^ mice with AAV expressing a Cre recombinase under the control of CaMKII promoter (AAV-CaMKII-Cre-GFP). Control AAVs expressed only GFP (AAV-CaMKII-GFP). AAV-CaMKII-Cre-GFP and control viruses were bilaterally injected either in the amygdala (CUMS-Amy group), or the medial prefrontal cortex (CUMS-mPFC group), or the nucleus accumbens (CUMS-NAc group), or the hippocampus (CUMS-Hip group). Three weeks after surgery, all the groups of mice were exposed to the CUMS protocol. The GFP group of mice was injected in one of the four aforementioned brain regions with AAV-CaMKII-GFP, but not exposed to CUMS. For more clarity see supplementary figure 1b. First, we verified the correct coordinates of the injection sites (supplementary figure 2a), and the effects of to the local deletion of the *PTK2B* gene on reduction of Pyk2 protein levels in the injection site by immunofluorescence (supplementary figure 2b–e). Our results showed a clear decrease or disappearance of Pyk2 immunoreactivity in the AAV-Cre-targeted brain regions.

**Fig. 2:**
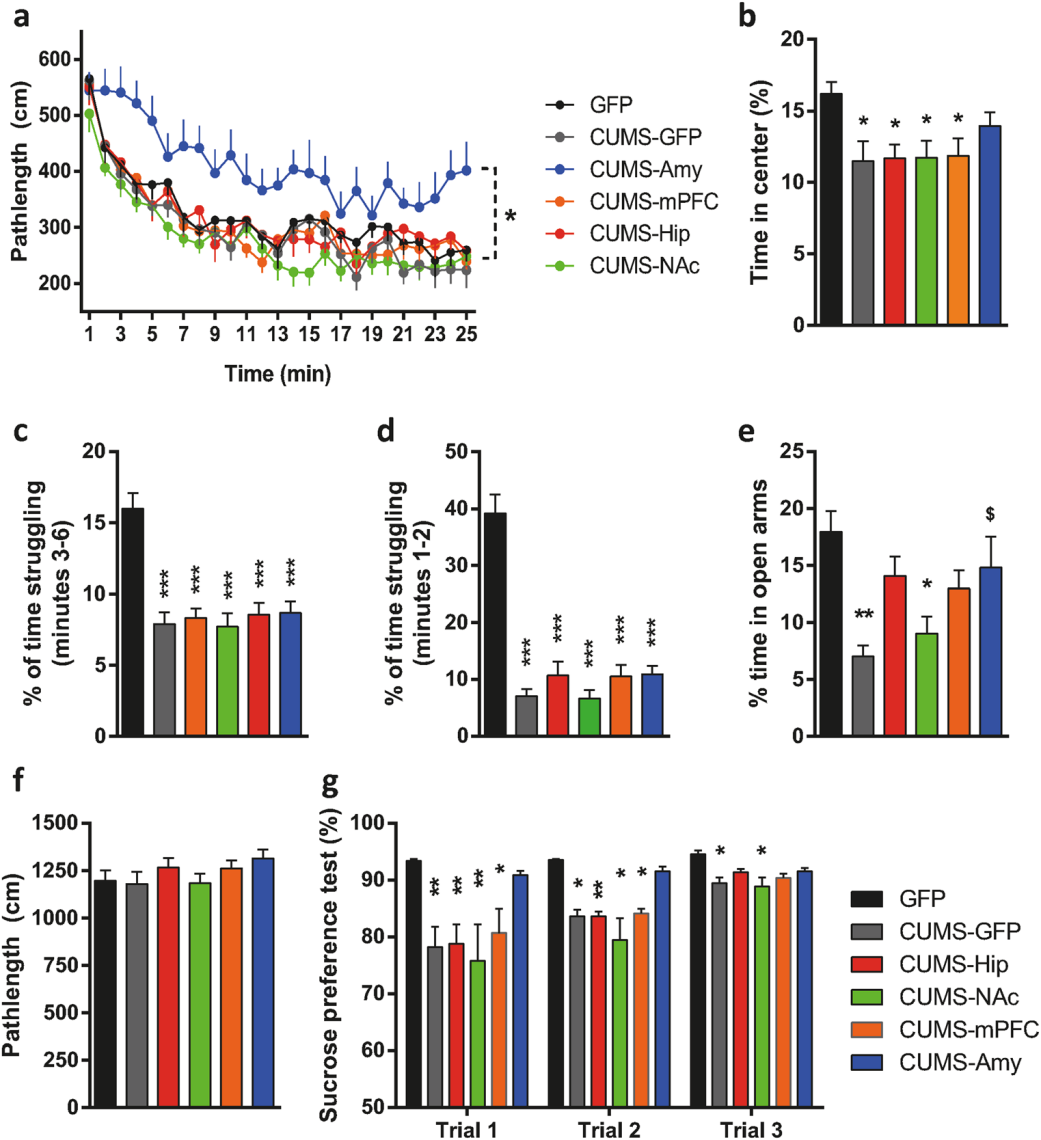
Consequences of the regional-specific ablation of Pyk2 on the behavioral effects of CUMS. **a** Pathlength traveled in an open field apparatus during a 30 min session. Two-way ANOVA, genotype effect: *F*_(5, 74)_ = 5.42, *p* = 0.0003. **b** Time in the center in an open field apparatus during 30 min. One-way ANOVA, genotype effect: *F*_(5, 76)_ = 2.349, *p* = 0.0488. **c** Forced swimming test: % of time struggling from the 3rd to the 6th minute in a 6-min long session. One-way ANOVA: *F*_(5, 76)_ = 20.83, *p* < 0.0001. **d** Forced swimming test: % of time struggling from the 1st to the 2nd minute in a 6-min long session. One-way ANOVA: *F*_(5, 76)_ = 45.34, *p* < 0.0001. **e** Elevated plus maze test, time spent in open arms, One-way ANOVA and Tukey’s post hoc test: *F*_(5, 76)_ = 4.396, *p* = 0.001. **f** Pathlength traveled in the elevated plus maze. One-way ANOVA and Tukey’s post hoc test: *F*_(5, 76)_ = 1.19, *p* = 0.32. **g** Sucrose preference test: Preference for sweet water over plain water (%). Three consecutive days of testing were evaluated in the sucrose preference test. One-way ANOVA: day 1, *F*_(5, 79)_ = 2.91, *p* = 0.018; day 2: *F*_(5, 79)_ = 7.34, *p* < 0.0001; day 3: *F*_(5, 79)_ = 3.86, *p* = 0.003. Post-hoc Tukey’s test. Data are means + SEM. **a–g** GFP group (*n* = 10); CUMS-GFP group (*n* = 13); CUMS-mPFC group (*n* = 15); CUMS-Nacc group (*n* = 15); CUMS-Hipp group (*n* = 15); CUMS-Amy group (*n* = 14). *p < 0.05 and **p < 0.01 compared to unstressed GFP group and ^$^*p* < 0.05 compared to CUMS-GFP group

Next, after 28 days of CUMS all groups of mice were evaluated in a battery of behavioral tests namely open field test, plus maze test, forced swimming test, and sucrose preference test. Concerning the general locomotor activity, all groups of mice habituated well and showed equal levels of locomotor activity in the open field except for the CUMS-Amy group which showed increased pathlengths but normal habituation (Fig. 2a). Regarding to the parameter time in the center, all the groups of mice displayed reduced time in the center of the arena when compared with the GFP group with the exception of the CUMS-Amy group (Fig. 2b). We then used the forced swimming test in all the groups (Fig. 2c). Regardless of the regional deletion, all groups of mice exposed to CUMS struggled much less than the unstressed GFP group confirming that the changes in the levels of Pyk2 in any tested brain region had no effect on the performance in the forced swimming test. We also analyzed the two first minutes of the assay and again all groups of mice exposed to CUMS struggled much less than the unstressed GFP group (Fig. 2d). Then, the elevated plus maze was performed in all groups of mice (Fig. 2e). As compared to all the other groups, the CUMS-GFP group spent the lowest time in the open arms. The CUMS-NAc group displayed reduced time in the open arms similar to those of CUMS-GFP mice. In contrast, the CUMS-Amy group had levels of open arms exploration close to those of unstressed GFP controls, and significantly different from those of the CUMS-GFP group. Finally, the CUMS-mPFC and CUMS-Hip groups showed intermediate alterations regarding to the time spent in open arms. These results did not appear to depend on general locomotor activity in the plus maze as shown in Fig. 2f. This was a discrepancy from the CUMS-Amy group when comparing the locomotor activity parameter in the elevated plus maze and in the open field test. The CUMS-Amy group showed hyper locomotion only in the latter. This discrepancy with the elevated plus maze is likely due to the fact that the open field is more useful for locomotor measurements than it is to anxiety and elevated plus maze is more useful for anxiety-like measurements than it is to locomotor behavior^26,27^. Altogether, these results indicate that the CUMS-Amy group was the less affected by the CUMS protocol in parameters evaluated by the elevated plus maze and open field test. Finally, we tested in all six groups of mice their preference for sweet (2% sucrose) or plain water, as in Fig. 1h (Fig. 2g). As expected, control GFP mice exhibited the highest level of preference towards palatable water in the presence of both plain and sucrose-water. In contrast, although the CUMS-GFP mice had some preference for sweet water, this preference was significantly reduced as compared to GFP CUMS-naive controls. CUMS-mPFC, CUMS-Hip, and CUMS-NAc displayed similarly low levels of sucrose preference as compared to CUMS-GFP and significantly reduced preference when compared with the GFP group during the first 2 days of testing. Interestingly the CUMS-Amy group was the only group showing indistinguishable levels of sucrose preference as compared to GFP mice indicating that CUMS did not affect the preference towards the sweet water in this group of mice. In summary, the present results show that the reduced sequelae displayed by full Pyk2^−/−^ knockout mice after the CUMS protocol was completely mimicked when Pyk2 expression was specifically removed in the amygdala.

### Pyk2 levels and its activation are not altered by stress in the amygdala

**Fig. 3:**
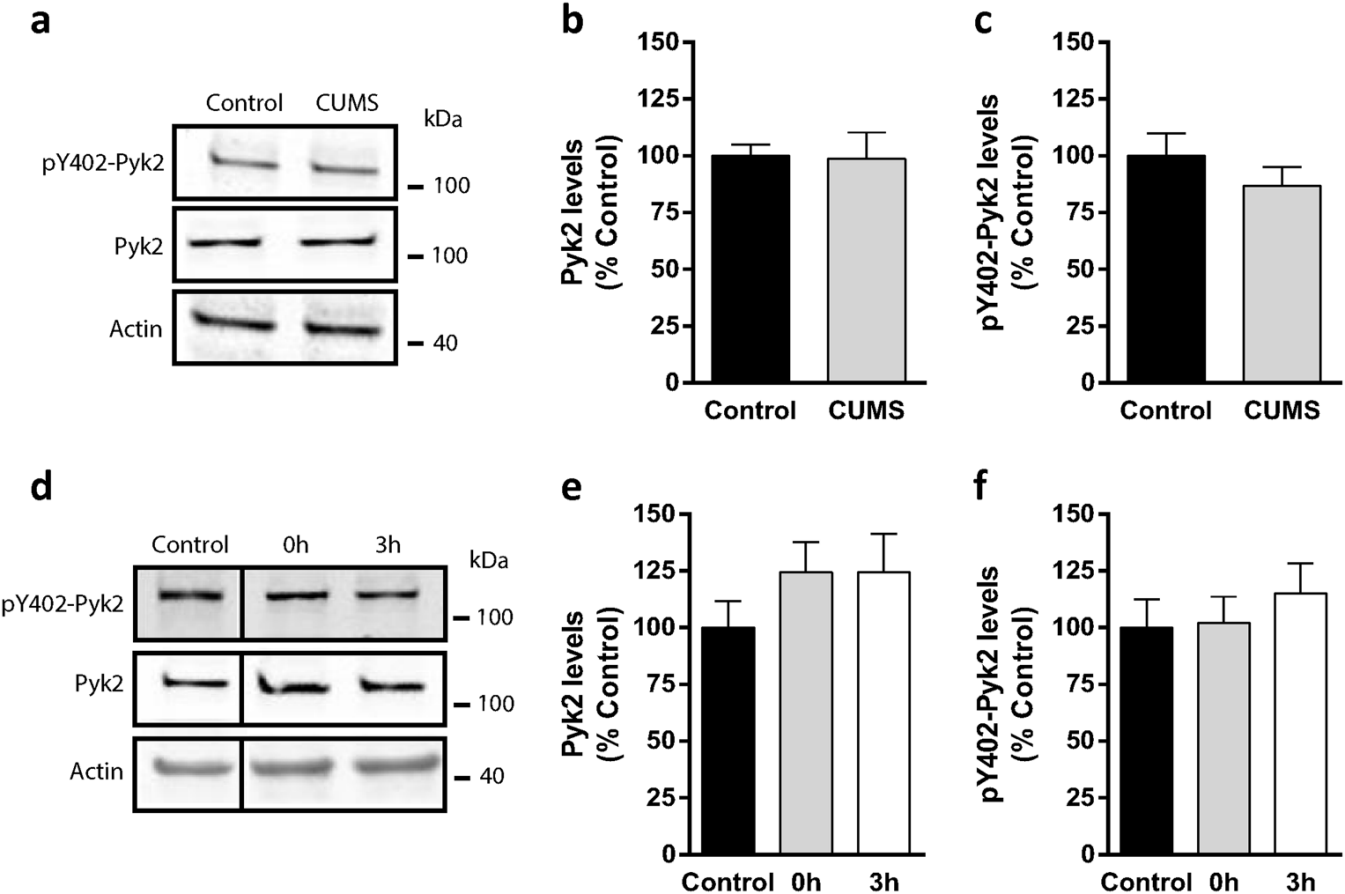
Lack of effect of stress on the total levels and phosphorylation of Pyk2. **a–c** Wild type mice subjected or not to a 28-day CUMS protocol (from Fig. 1) and sacrificed 10 days later (after the behavioral assessment was ended). **a** Immunoblotting analysis of Pyk2 and its activated form autophosphorylated on Tyr402 (pY402-Pyk2) and tubulin as a loading control. **b** Densitometric quantification of total Pyk2 as in **a**. Two-tailed Student’s *t*-test: *t*_17_ = 1.24, *p* = 0.23. **c** Densitometric quantification of pY402-Pyk2 as in **a**. Two-tailed Student’s *t*-test: *t*_17_ = 1.31, *p* = 0.19. In **b** and **c** data were normalized to tubulin for each sample and expressed as percentage of Pyk2^+/+^ controls. **d–f** Another set of wild type mice were subjected or not to acute restraint stress and sacrificed immediately (0 h) or 3 h later. **d** Immunoblotting analysis of Pyk2, pY402-Pyk2, and tubulin as a loading control. **e** Densitometric quantification of total Pyk2 results as in **d**. One-way ANOVA: *F*_(2, 14)_ = 0.88, *p* = 0.43. **e** Densitometric quantification of pY402-Pyk2 as in **d**. One-way ANOVA: *F*_(2, 14)_ = 0.44, *p* = 0.66. Data are means + SEM. Tukey’s post hoc test was used in **e** and **f**. In **b**, **c**, **e**, and **f**, data were normalized to tubulin and expressed as percentage of Pyk2^+/+^ unstressed controls. In **a**–**c** *n* = 9 mice/group; in **d**–**f** *n* = 5–6 mice per group. In **a** and **d** molecular weight markers position is indicated in kDa

To address the molecular mechanisms underlying the phenotype showed by the mice lacking PyK2 in the amygdala, we measured Pyk2 total protein levels and its phosphorylation at Tyr-402, the autophosphorylation site^28^, a good indicator of its kinase activity, in the amygdala of Pyk2^+/+^ mice subjected to 28 days of CUMS vs. naive control Pyk2^+/+^. When animals were sacrificed 24 h after the end of the CUMS protocol, we did not observe any change either in the total levels of Pyk2 (Fig. 3a, b) nor in phosphoTyr-402-Pyk2 (Fig. 3a, c). We then hypothesized that the changes in the total or phospho-Pyk2 levels might be more acute and/or transient or that they could take place earlier after the exposure of the mice to stress. Thus, we designed a new experiment in which Pyk2^+/+^ mice were acutely stressed by subjecting them to an hour restrain. Mice were sacrificed 0 or 3 h after the restrain protocol and the amygdala was quickly dissected out. Unstressed Pyk2^+/+^ mice were used as controls. Again, no change in either total Pyk2 (Fig. 3d and e) or phosphoTyr-402-Pyk2 (Fig. 3d, f) was observed in the stressed Pyk2^+/+^ mice as compared to control mice. In summary, these results show that neither acute stress nor CUMS alter Pyk2 levels or autophosphorylation in a manner that is detectable at the tissue level.

### Excitatory synapses in principal neurons of the basolateral amygdala (BLA) are increased during CUMS in Pyk2^+/+^ but not in Pyk2^−/−^ mice

**Fig. 4:**
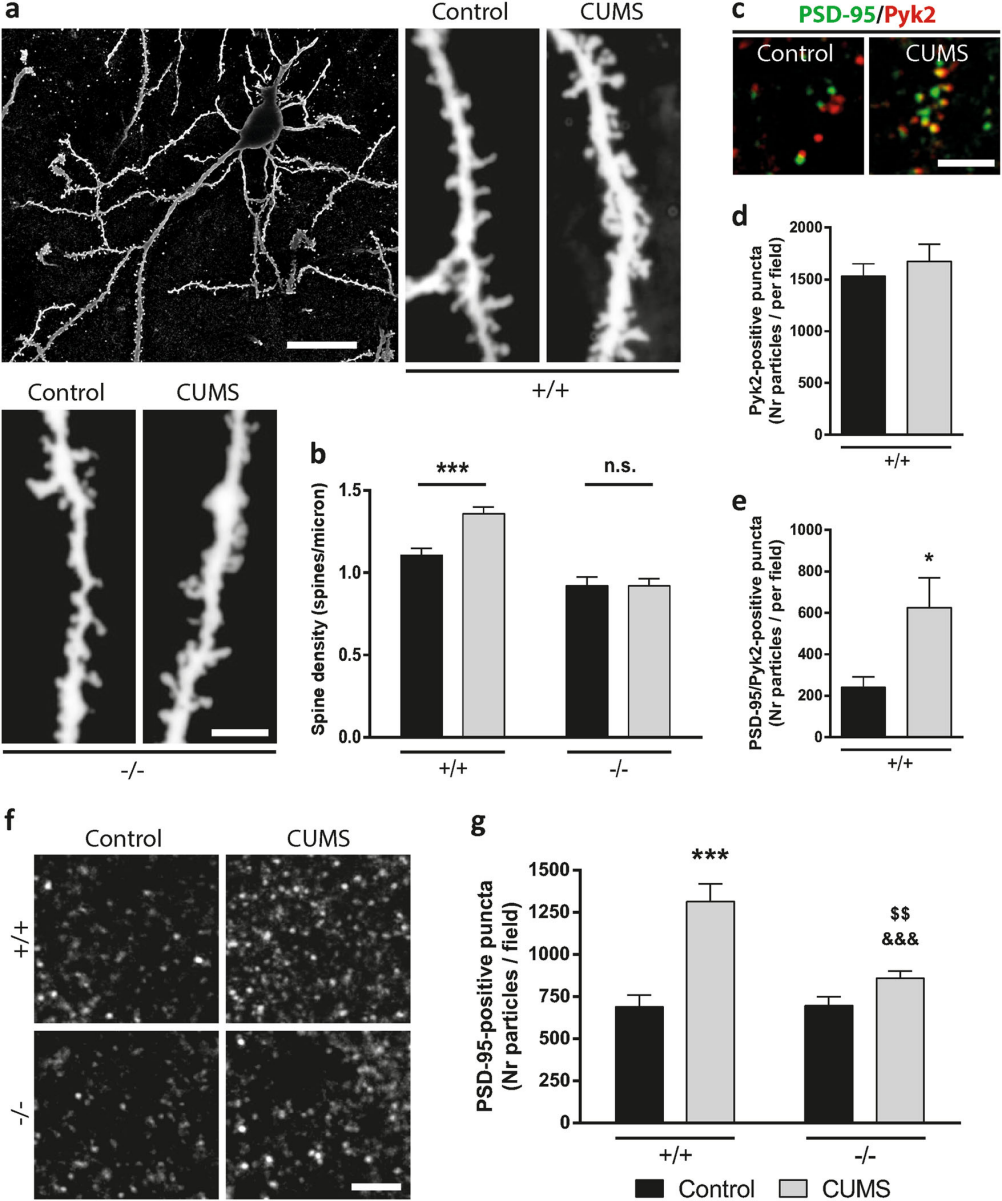
Pyk2 knockout prevents the changes in dendritic spines and PSD-95 induced by chronic stress in the amygdala. **a** Golgi-Cox-stained apical dendrites of a basolateral amygdala principal neuron from Pyk2^+/+^ and Pyk2^−/−^ unstressed control mice and in Pyk2^+/+^:CUMS and Pyk2^−/−^:CUMS mice. The images are negatives for better visualization. Scale bars, 15 and 3 µm, respectively. **b** Quantification of spine density in dendrites as in **a**. Two-way ANOVA, interaction, *F*_(1, 261)_ = 17.92, *p* < 0.0001, Bonferroni’s post hoc test (55–60 dendrites from 5 mice per group). **c** Confocal images of the basolateral nucleus of the amygdala sections from Pyk2^+/+^ controls and Pyk2^+/+^ mice after CUMS immunolabelled for PSD-95 (green) and Pyk2 (red). We analyzed 1 image per slice, three slices per mouse, seven mice per group. Scale bar, 4 µm. **d** and **e** Quantification of results as in **c**. **d** Number of Pyk2-positive puncta per field, Student’s *t*-test; *t* = 0.69, *p* = 0.5. **e** Number of Pyk2/PSD-95-double-positive puncta per field, Student’s *t*-test, *t* = 2.52, *p* = 0.026. **f** PSD-95-positive puncta in the basolateral nucleus in Pyk2^+/+^ and Pyk2^−/−^ control mice and in Pyk2^+/+^:CUMS and Pyk2^−/−^:CUMS mice. Scale bar, 3 μm. **g** Quantification of PSD-95-positive puncta in basolateral nucleus as in **f**. Two-way ANOVA, interaction, *F*_(1, 33)_ = 9.53, *p* < 0.004; stress effect *F*_(1, 33)_ = 27.71, *p* < 0.0001, Bonferroni’s post hoc test (1 image per slice, three slices per mouse, 8–9 mice per group). Data are means + SEM. ***p < 0.001 compared to Pyk2^+/+^ unstressed controls, ^&&&^*p* < 0.001 compared to Pyk2^+/+^:CUMS, and ^$$^*p* < 0.01 compared to Pyk2^−/−^ controls

**Fig. 5:**
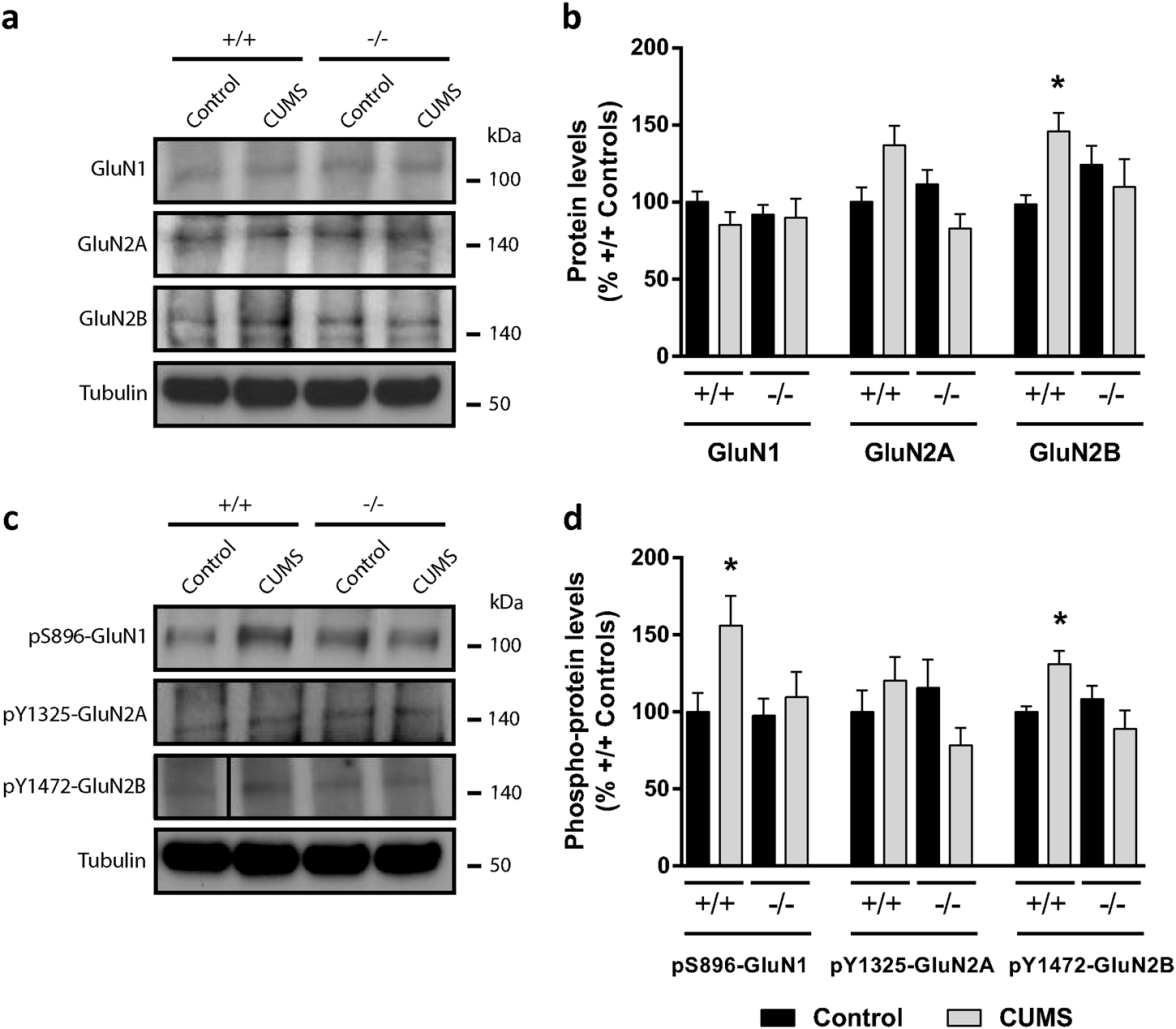
Pyk2 knockout prevents the changes in NMDA receptors levels and phosphorylation induced by chronic stress in the amygdala. NMDARs were studied by immunoblotting in Pyk2^+/+^ and Pyk2^−/−^ mice unstressed (Control) or 10 days after being subjected to a CUMS paradigm. **a** Immunoblotting analysis of GluN1, GluN2A, GluN2B, and tubulin as a loading control. **b** Densitometric quantification of results as in **a**. Two-way ANOVA interaction, GluN1, *F*_(1, 30)_ = 0.33, *p* = 0.95, GluN2A, *F*_(1, 30)_ = 1.14, *p* = 0.70, GluN2B, *F*_(1, 30)_ = 6.04, *p* = 0.02, Tukey’s post hoc test. **c** Immunoblotting analysis of pS896-GluN1, pY1325-GluN2A, pY1472-GluN2B, and tubulin as a loading control. **d** Densitometric quantification of results as in **c**. Two-way ANOVA interaction, pS896-GluN1, *F*_(1, 29)_ = 5.75, *p* = 0.02; pY1325-GluN2A, *F*_(1, 29)_ = 0.42, *p* = 0.51, pY1472-GluN2B, *F*_(1, 29)_ = 7.35, p = 0.01, Tukey’s post hoc test. Data are means + SEM. In all experiments *n* = 8–9 mice per group. From **a** to **d**: *p < 0.05 compared to Pyk2^+/+^ unstressed controls. In **a** and **c** molecular weight markers position is indicated in kDa

Pyk2 has functions that are dependent on its kinase activity and others that are more related to other properties, such as scaffolding^15,18,19^. In our previous work we observed that the decreased spine density in Pyk2^−/−^ hippocampal neurons is rescued by Pyk2 re-expression independently of its kinase activity^18^. Chronic stress and subsequent depression-like phenotype correlate with an increase in dendritic spines in the principal neurons of the BLA nuclei^8^. We therefore investigated whether the lack of Pyk2 in the amygdala could prevent the formation of these new dendritic spines. We used Golgi staining in a sub-set of Pyk2^+/+^, Pyk2^−/−^ control mice, as well as in Pyk2^+/+^:CUMS and Pyk2^−/−^:CUMS mice that were sacrificed 24 h after the last day of CUMS to study the spine density in the principal projection neurons of the BLA. First, our analysis confirmed the previous reports showing that dendritic spine density was significantly increased in Pyk2^+/+^ mice, 10 days after the CUMS protocol, as compared to Pyk2^+/+^ controls (Fig. 4a, b). In contrast, this stress-induced effect was completely absent in Pyk2^−/−^:CUMS mice compared to Pyk2^-/-^ control mice (Fig. 4a, b). To further explore the role of Pyk2 we performed a double immunofluorescence in Pyk2^+/+^ and Pyk2^+/+^:CUMS mice to study whether Pyk2 translocates and colocalizes with PSD-95 after chronic stress as previously published in stimulated pyramidal neurons of the hippocampus^15^. CUMS induced a clear increase in Pyk2 and PSD-95 colocalization (Fig. 4c–e). We then evaluated the PSD-95 dynamics in response to CUMS and counted the number of PSD-95 clusters, which correspond to post-synaptic densities, in the BLA of wild type and Pyk2^−/−^ mice. In Pyk2^+/+^ mice CUMS increased the number of PSD-95-positive clusters per field as compared to Pyk2^+/+^ controls (Fig. 4f, g). In Pyk2^-/-^ mice the CUMS protocol also increased the number of PSD-95-positive particles, but this effect was significantly less pronounced as compared to Pyk2^+/+^:CUMS mice (Fig. 5f, g) confirming the hypothesis that the increase in PSD-95 clusters due to chronic stress is significantly weaker in Pyk2^−/−^ mice.

### N-methyl-d-aspartate receptors (NMDARs) alterations in the amygdala after CUMS are Pyk2-dependent

The increase of Pyk2/PSD-95 colocalization in the BLA after CUMS was coincident with both the clustering of PSD-95 at excitatory synapses and the increase in spine density in the BLA. We therefore hypothesized that the increase in the post-synaptic density scaffold could correspond to changes in the NMDARs in the principal neurons of the BLA. To test this hypothesis, we evaluated by immunoblotting the total and phosphorylated levels of several NMDAR subunits in the amygdala of Pyk2^+/+^:CUMS and Pyk2^-/-^:CUMS mice and we compared the results with those obtained in Pyk2^+/+^ and Pyk2^−/−^ controls. First, total NMDA receptors GluN1 and GluN2A levels were not changed in mice subjected to CUMS as compared to control mice (Fig. 5a, b). In contrast, we observed an increase in GluN2B total protein levels in Pyk2^+/+^:CUMS as compared to Pyk2^+/+^ mice. The increase in GluN2B was negligible when comparing Pyk2^−/−^:CUMS with Pyk2^−/−^ mice. The phosphorylated forms, pS896-GluN1 and pY1472-GluN2B were also increased in Pyk2^+/+^:CUMS compared to Pyk2^+/+^ control mice (Fig. 5c, d). When we calculated the ratio pY1472-GluN2B/GluN2B the increase disappeared (data not shown) indicating that increased pY1472-GluN2B levels were due to a general up-regulation of the GluN2B protein subunit. In Pyk2^-/-^:CUMS mice however, these changes were completely abolished when compared to Pyk2^−/−^ mice. These results indicate that, in wild type mice, CUMS induced a long-lasting change in the properties of excitatory synapses in the BLA, which was apparent as an increase in the Ser896 phosphorylation of GluN1 and in the GluN2B protein levels. In contrast these changes were prevented in the absence of Pyk2, revealing the key role of this protein in synaptic adaptations in response to chronic stress.

## Discussion

Here, we show the role of Pyk2 in the response to a model of chronic stress. We showed that although in basal conditions (i.e. in the absence of stress) the absence of Pyk2 does not modify the responses in anxiety-like and depression-like behavioral tests, PyK2 ablation in the adult confers a marked resistance to the sequelae induced by CUMS, as shown by the normal performances in the plus maze and sucrose preference tests. We also identified the amygdala as a core structure in the modulation of the observed phenotype in Pyk2-deficient mice. Importantly, we provide evidence for a key role of Pyk2 in the regulation of dendritic spine density in the projection neurons of the BLA, with a concomitant PSD-95 stabilization at synapses. Accordingly, the modifications of NMDARs were prevented in the amygdala of Pyk2-deficient mice exposed to the CUMS paradigm. The ensemble of those results strongly indicates a role of Pyk2 in regulating the structural plasticity upon chronic stress.

Interestingly, we observed a dissociation in the Pyk2-dependence of different components of the behavioral alterations induced by CUMS. We observed that in full Pyk2 knockout mice the CUMS protocol failed to induce a reduction of sucrose preference, as well as a decrease on the exploratory behavior in the open arms of the elevated plus maze. In order to identify the regions mainly implicated in the reduced alterations induced by CUMS we focused on the brain areas showing the highest levels of Pyk2 expression, and that are associated to chronic stress and major depression: medial prefrontal cortex, nucleus accumbens, hippocampus, and amygdala^8,13,20,29–31^. We identified the amygdala as the brain region mostly implicated in the absence of alterations (detected by the plus maze and sucrose preference tests) after CUMS in Pyk2-deficient mice. In other words, Pyk2 deletion in amygdala neurons was sufficient to prevent the sequelae induced by the CUMS paradigm.

It is well known that a history of repeated or prolonged stress often leads to hyperactivity of the amygdala^32–34^. Furthermore, human studies show that depression and anxiety are also associated with amygdala hyperactivity^35–38^. In animal models, the BLA has emerged as a target for the effects of repeated stress in a range of anxiety and depressive behaviors. Furthermore, these behavioral abnormalities are associated with hyperactivity of BLA neurons resulting from an increased excitatory input to those neurons^39–43^. The increase in the excitatory synaptic inputs to BLA after repeated stress correlates with an increase in dendritic spines and dendritic length^44–46^. Pyk2, is a tyrosine kinase involved in both the induction and maintenance of synaptic plasticity, in the regulation of dendritic spines density and morphology, and in the formation of memory traces^17,18^. Our results show that Pyk2 plays an important role in the structural plasticity induced by the CUMS paradigm in the neurons of the BLA, including the increase in dendritic spines. Importantly, the amygdala can sense a stressful event and generate an appropriate response to it; however, the dysfunction of this mechanism might lead to the development of a major depression^31^. Thus, our hypothesis is that the inactivation of Pyk2, principally in the amygdala, would lead to the alteration of the processing of the stressful stimuli turning the animal “resistant” to anhedonia-like and anxiety-like phenotypes.

Regarding to the unchanged levels of phosphorylation of Pyk2 in the amygdala after CUMS or acute stressful stimuli, we hypothesize that there could be different interpretations. First, Pyk2 could be transiently activated in few dozens of minutes following stress and return to a basal activity after 1 h. If so, our methods were unsuitable for the detection of such activity. A second interpretation is that the role of Pyk2 that we observed in the stress-activated pathway could rely on the phosphorylation/dephosphorylation of another residue than the Tyr-402. Our group has already shown in hippocampal slices that depolarization induces a calcineurin-dependent nuclear translocation of Pyk2 involving the dephosphorylation of Ser-778^47^. Finally, one can also hypothesize that the function of Pyk2 in response to stress does not need an increased activation of itself compared to basal level. If so, we can imagine the involvement of interactors of Pyk2 which regulate its substrate specificity or limit its accessibility towards a subset of its substrates by anchoring Pyk2 in a specific subcellular localization. As an example, cyclin-dependent kinases (CDKs) perfectly illustrate this type of regulation. Interaction of CDKs with cyclins can help to target their activity toward precise substrates, or to limit their accessibility by shuttling them to a cellular compartment, such as the Golgi apparatus or nucleus^48^. In this line, we hypothesize that there is a necessary role of Pyk2 at synapses without a need for an increased activation. It can also be related to kinase-independent functions of Pyk2, which we previously showed to be sufficient for regulating spine number in hippocampal neurons in culture in response to glutamate stimulation^18,19^. In the hippocampus Pyk2 interacts with PSD-95^15,49^. We did show that Pyk2 stabilizes PSD-95 in the excitatory synapses and regulates synaptic plasticity^18^. PSD-95 anchors essential proteins, including NMDARs, which are implicated in the regulation of excitatory synapses plasticity^50^, and in the control of the number and morphology of dendritic spines^51^. We showed an increased Pyk2/PSD-95 colocalization in the amygdala of the chronically stressed mice concomitant with an increase in the number of puncta positive for PSD-95. We also observed an increase in GluN2B and pSer896-GluN1 induced by CUMS and prevented in the absence of Pyk2. Since Pyk2 is a tyrosine kinase, which cannot be directly responsible for Ser896-GluN1 phosphorylation, these changes rather indicate the role of Pyk2 in the maintenance or function of excitatory synapses during the processes described here. The role of Pyk2 is likely to be, in part, related to its interaction with PSD-95.

Supporting our results, several studies showed that PSD-95 is essential for the maintenance of long-lasting fear memories via remodeling excitatory synapses^52^ and that both NMDARs subunits and PSD-95 are increased in the amygdala of postmortem samples from patients with MDD^53^. The increase in GluN1 Ser896 phosphorylation and in the number of GluN2B subunits phosphorylated on Tyr1472 we observed after CUMS suggests an enhanced NMDAR function with increased channel opening probability, calcium influx, and receptor recruitment to the membrane^54^. NMDAR was shown to play an essential role in the aforementioned hyperactivation of the amygdala upon chronic stress and in the subsequent formation of new excitatory synapses^55,56^. Since the synaptic changes in the amygdala were prevented in the absence of Pyk2, this kinase appears to be a core molecule mediating the effects of chronic stress.

Intriguingly, we show that the Pyk2-deficient mice behaved as the non-stressed controls in the sucrose preference test but not in the forced swimming test. It is known that a missing response to a reward stimulus is the behavioral correlate of the anhedonia, a core clinical symptom of the depression. When looking at our results we should consider that, although the amygdala is widely accepted as important for the recognition of negative emotions such as fear^57,58^, several works in animal models, as well as in humans point out the involvement of this region in processing of positive emotions^59,60^. There is evidence that the amygdala has a role in the processing stimulus—reward learning by interacting with the cortico- limbic and meso-limbic systems. This is particularly true for the lateral amygdala that seems to be especially important for the association of a value to the reward. For example, in animals lesioned in basolateral but not central amygdala^61^ we can prevent the normal loss of the response to a stimulus predicting a food reward when paired to a malaise-induced injections of lithium chloride^62^. We could hypothesize that one possible explanation of our results could be a preferential effect of the deletion of PTK2B in the amygdala in tests tracking the effect of chronic stress of more reward-related symptoms rather than measuring the behavioral despair, that in the other hand could be more related to other regions of the brain. Partially supporting this idea one study showed that in rodents, the level of 5-HT release following a forced swim test is reduced in hippocampus, nucleus accumbens, and cortex but not in the amygdala^63^. However, the most obvious explanation for this lack of changes in the forced swimming test is that Pyk2 might not be important for its principal and underlying molecular/physiological processes. Another divergence in our work comes from the CUMS-Amy group when comparing the locomotor activity parameter in the elevated plus maze and in the open field test. The CUMS-Amy group showed hyper locomotion only in the latter. This discrepancy with the elevated plus maze is likely due to the fact that the open field is more useful for locomotor measurements than it is to anxiety-like behaviors and elevated plus maze is more useful for anxiety-like measurements than it is to locomotor behavior^26,27^. Thereby, intrinsic differences of the apparatus make the open field test more sensitive to changes in locomotor activity than elevated plus maze. Accordingly, other discrepancies between the open field and the elevated plus maze have been previously reported^64–67^. Finally, we found that mice with deletion in the medial prefrontal cortex and hippocampus showed decreased preference for sweet water in the first trials of the sucrose preference test, but they finally became unaffected in the trial number 3. One possible reason is that the two regions seem to contribute together to the short-term memory^68^. Indeed, we already showed in a previous paper that Pyk2 is involved in the regulation of memories mediated by the hippocampus^18^. Therefore, we hypothesize that deletion of Pyk2 in the hippocampus or the medial prefrontal cortex could induce mnemonic alterations that would lead to a faster habituation/acceptation of the sweet solution.

In conclusion, recent large-scale human genetic studies have identified *PTK2B* as a gene associated with neuroticism^22^ which in turn is a risk factor to develop MDD^23^. Here we propose for the first time a potential mechanism that could explain the human association of *PTK2B* to the risk to develop depression after chronic stress. Our results reveal a new role of Pyk2 in neuronal dysfunction. We show that it is necessary for several components of the depressive-like phenotype, which appears to require Pyk2-dependent spine alterations in the amygdala. Thus, Pyk2 could be included in the list of molecules known as negative modulators of resilience-like phenotypes against chronic stress and depression, such as BDNF and ΔFOSB^30^. Our findings indicate that targeting Pyk2 in patients suffering from multiple and chronic stressful psychological insults could help to prevent the associated depressive symptoms. Since the activation state of Pyk2 in the amygdala is not clearly altered in response to stress, it could be particularly attractive to modulate the structural role of Pyk2 without affecting its activity. Such structural mechanism was already evoked in our previous paper^18^ and is reminiscent of that reported for the closely related focal adhesion kinase (FAK) in non-neuronal cells^69,70^. This alternative signaling may be linked to scaffolding properties of Pyk2 and/or its interaction with specific partners. For instance, in our last paper, we showed that although dendritic spines density was altered in Pyk2-deficient neurons, it could be rescued by transfecting either a mutant form of Pyk2 with a point mutation of the autophosphorylation site or a kinase-dead Pyk2 with a K457A mutation. However, transfection of a mutant form of Pyk2 unable to bind to PSD-95 (Pyk2_1–840_) could not rescue the phenotype showing the importance of this interaction in this particular pathway, which does not involve the autophosphorylation of Pyk2. To sum up, Pyk2 may function as a scaffold which gather some proteins, independently of its kinase activity.

## Supplementary information

Supplementary methods and figures
