# Supplementary material for: Pyk2 in the amygdala modulates chronic stress sequelae via PSD-95-related micro-structural changes
Source: Transl Psychiatry. 2019 Jan 15;9:3. doi: 10.1038/s41398-018-0352-y (PMC6341095; doi:10.1038/s41398-018-0352-y)
Supplement: Supplementary file 1 — Supplementary methods and figures [file 41398_2018_352_MOESM1_ESM.docx]

**Supplementary methods**

*Animals*

For Pyk2 deletion, Pyk2^f/f^ C57Bl/6 mice were generated in which the *PTK2B* exons 15b-18 were flanked with LoxP sequences leading to a deletion that disrupts the protein kinase domain following Cre recombination^1, 2^. Pyk2^-/-^ mice and Pyk2^f/f^ mice were genotyped from a tail biopsy (Charles River, Saint-Germain-Nuelles, France) using for DNA amplification of floxed *PTK2B* as forward primer 50-GAGAGTGCTGGGTACTCCAGACTCAGATAG-30 and as reverse primer 50-TTCAGGAACACCAGAGAACTAGGGTGG-30 and previously reported primers^2^ for Pyk2^-/-^ mice. Housing room was kept at 19–22ºC and 40–60% humidity, under a 12:12 h light/dark cycle and mice had *ad libitum* access to food and water. Animal experiments and handling was in accordance with ethical guidelines of Declaration of Helsinki and NIH, (1985-revised publication no. 85-23, European Community Guidelines), and French Agriculture and Forestry Ministry guidelines for handling animals (decree 87849, license A 75-05-22) and by the local animal ethical committees (Darwin Committee at Sorbonne Université and 477/15 at the *Universitat de Barcelona*), in compliance with the Directive 2010/63/UE of the European Commission and Spanish decree RD 53/2013. Behavioral experiments were always carried out during the morning, in a 12h light/dark cycle.

*Chronic unpredictable mild stress (CUMS)*

One day before the beginning of the CUMS procedure the mice were individually housed and maintained isolated for the entire experiment. The CUMS procedure followed a random weekly schedule of commonly used mild stressors (one per day): restrain (1 h), food or water deprivation (24 h), home cage inclination (1 h), exposition to fox urine (20 min), social interaction with other animals independent of the experiment (30 min) and exposition to hairdryer (5 min). The CUMS protocol lasted 28 days.

*Open field*

The apparatus consisted of a white square arena measuring 40 x 40 x 40 cm in length, width and height. A dimly light intensity of 60 lux was detected throughout the arena. Animals were placed on the arena center and allowed to explore freely for 30 min. Spontaneous locomotor activity was measured. At the end of each trial, defecations were removed, and the apparatus was wiped with 30% ethanol. Animals were tracked and recorded with SMART junior software (Panlab, Spain).

*Elevated plus maze*

Briefly, the plus maze was made of plastic and consisted of two opposing 30 × 8 cm open arms, and two opposing 30 × 8 cm arms enclosed by 15 cm-high walls. The maze was raised 50 cm above the floor and lit by dim light. Each mouse was placed in the central square of the maze, facing an open arm and its behavior was scored for 5 min. At the end of each trial any defecation was removed, and the apparatus was wiped with 30% ethanol. We recorded the time spent in the open arms, which normally correlates with low levels of anxiety. Animals were tracked and recorded with SMART junior software (Panlab, Spain).

*Forced swimming test*

The forced swimming test was used to evaluate behavioral despair. Animals were subjected to a 6 min trial during which they are forced to swim in an acrylic glass (35 cm height x 20 cm diameters) filled with water, and from which they cannot escape. The time that the test animal spent in the cylinder without making any movements except those required to keep its head above water was measured.

*Sucrose preference test*

Sucrose preference testing was carried out in the animal’s home cage. Mice were presented with 2 dual bearing sipper tubes. One tube containing plain drinking water and the other a 2% sucrose solution. Two days before the beginning of the test, all mice were habituated to the presence of two drinking bottles containing just water in their home cage. Following this acclimation, mice have had the free choice between the 2% sucrose solution or plain water for a period of 3 days. Water and sucrose solution intake was measured daily, and the positions of two bottles was switched daily to reduce any side bias. Sucrose preference was calculated as a percentage of the volume of sucrose intake over the total volume of fluid intake per day.

*Viral constructs and stereotaxic injection*

For the specific deletion of Pyk2 in the various brain regions, 8-week-old Pyk2^f/f^ mice were stereotaxically injected with an adeno-associated virus (AAV) expressing the Cre recombinase (AV-9-PV2521, AAV9.CamKII.HI.eGFP-Cre.WPRE.SV40, AAV-Cre from Perelman School of Medicine, University of Pennsylvania, USA). As a control, we injected AAVs expressing GFP (AV-9-PV1917, AAV9.CamKII0.4.eGFP.WPRE.rBG, AAV-GFP) from the same source). Mice were anaesthetized with ketamine-xylazine (100 mg/kg), and bilaterally injected with AAV-GFP or AAV-Cre (~2.6x10^9^ GS per injection) in one of the following brain regions: dorsal hippocampus, from the bregma (millimetres); anteroposterior, –2.0; lateral, ± 1.5; and dorsoventral (two points), -1.3 and -2.1; nucleus accumbens, anteroposterior, 1.3; lateral, ± 1.2; and dorsoventral -4.5; basolateral amygdala, anteroposterior, -1.45; lateral, ± 2.8; and dorsoventral -4.75; medial prefrontal cortex, anteroposterior, 1.75; lateral, ± 0.35; and dorsoventral -3.00. AAV injection was carried out in 2 min. The needle was left in place for 5 min for complete virus diffusion before being slowly pulled out of the tissue. After 2 h of careful monitoring, mice were returned to their home cage for 3 weeks. All mice subjected to surgery that survived and showed no ethical and healthy problems (such as head inclination or >15% of body weight loss) were also evaluated for CUMS and behavioural characterization. Once the behavioural characterization was done, half of the brain was used to verify the site of injection by immunofluorescence (see “tissue preparation and immunofluorescence” section). Mice that showed no viral transduction in the basolateral amygdala were excluded from the entire study. In total 6 mice were excluded following this criteria.

*Behavioral experimental design* *in Pyk2^f/f^ mice*

For the experiment of Pyk2 genetic deletion in different brain regions of
Pyk2^f/f^ mice (Fig. 2) we performed as follows: Six experimental groups of mice were generated by bilaterally injecting AAV expressing GFP (AAV-GFP) or GFP-Cre (AAV-Cre) in the hippocampus (Hip), nucleus accumbens (NAc), amygdala (Amy) or medial prefrontal cortex (mPFC) of Pyk2^f/f^ mice. Naïve control mice were injected with AAV-GFP in one of the four brain regions and not submitted to CUMS (GFP group), All other groups were subjected to CUMS: mice injected with AAV-GFP in one of the four brain regions (CUMS-GFP group), mice injected with AAV-Cre one of the four brain regions (CUMS-mPFC, CUMS-NAc, CUMS-Hip, and CUMS-Amy groups). After CUMS, all groups (including the naive unstressed group) were subjected to a set of behavioral paradigms, as in Fig. 1, to evaluate anxiety, behavioral despair and anhedonia. A graphic summary is showed in supplementary figure 1b.

*Tissue preparation and immunofluorescence.*

Mice were deeply anaesthetized (pentobarbital, 60 mg/kg i.p.) and intracardially perfused with 40 g/l paraformaldehyde solution in 0.1 M sodium phosphate, pH 7.2. Brains were removed and post-fixed overnight in the same paraformaldehyde solution. The brains were then sliced in a Vibratome (Leica, VT1000). All the following steps were done with gentle shaking. 40 µm thick coronal sections were washed three times in PBS, permeabilized in PBS-T (+3 µl/ml Triton X-100 and 30 µl/ml normal goat serum (Pierce Biotechnology, Rockford, IL, USA) for 60 min at room temperature, and washed three times. Brain slices were incubated overnight at 4ºC in the presence of primary antibodies in PBS-T: rabbit Pyk2 antibody (1:500, Sigma #07M4755, Chemical Co.,St Louis, MO, USA), mouse anti-PSD-95 1:500 (#QA210648, Thermo Scientific, MA, USA). Sections were then washed three times and incubated for 2 h at room temperature with fluorescent secondary antibodies: Cy3 goat anti-rabbit (1:200) and/or AlexaFluor 488 goat anti-mouse (1:200; both from Jackson ImmunoResearch, West Grove, PA, USA). No signal was detected in control sections incubated in the absence of the primary antibody.

*Confocal imaging and analysis.*

Immunostained tissue sections (30-µm thick) containing basolateral nuclei of the amygdala were imaged using a Leica Confocal SP5-II (63x numerical aperture lens, 5x digital zoom, 1-Airy unit pinhole). At least three slices per mouse, were analyzed, and up to three representative basolateral nuclei images were obtained from each slice. Four frames were averaged per z-step throughout the study. Confocal z-stacks were taken at 1,024 x 1,024 pixel resolution every 2 µm. The labelled PSD95-positive clusters number was quantified with NIH ImageJ freeware (Wayne Rasband, NIH) as described^2^.

*Golgi staining and spines analysis.*

Fresh brain hemispheres were processed following the Golgi-Cox protocol as described elsewhere^2, 3^. Briefly, mouse brain hemispheres were incubated in the dark for 14–17 days in filtered dye solution (K_2_Cr_2_O_7_, HgCl_2_ and K_2_CrO_4_ 50 g/l each). The tissue was then washed 3 x 2min in water and 30 min in 90% EtOH. 200-µm thick sections were sliced in 70% EtOH on a vibratome (Leica) and washed in water for 5 min. Next, brain slices were reduced in 160 ml/l ammonia solution for 1 h before being washed in water for 2 min and fixed in 500 g/l Na_2_S_2_O_3_ for 7 min. After a 2-min final wash in water, sections were mounted on superfrost coverslips, dehydrated for 3 min in 50%, then 70, 80 and 100% EtOH (vol/vol), incubated twice for 5 min in a 2:1 isopropanol: EtOH mixture, followed by 5 min incubation in pure isopropanol and 2 x 5 min incubation in xylol. Bright-field images of Golgi-impregnated projecting spiny neurons of the basolateral nuclei of the amygdala were captured with a Nikon DXM 1200F digital camera. Image z-stacks were taken every 0.2 µm, at 1,024 x 1,024-pixel resolution, yielding an image with pixel dimensions of 49.25 x 49.25 µm. Only spines arising from the lateral surfaces of the dendrites were included in the study; spines located on the top or bottom of the dendrite surface were ignored. The total number of spines was obtained using the cell counter tool in the ImageJ software. At least 60 dendrites per group from at five mice per genotype were counted.

*Immunoblot analysis.*

Animals were sacrificed by cervical dislocation. The brain was removed and the amygdala was rapidly dissected on ice, and stored at -80ºC until use. Briefly, the tissue was lysed by sonication in 250 µl of lysis buffer (PBS, 10 ml l^-1^ Nonidet P-40, 1mM PMSF, 10 mg l^-1^ aprotinin, 1mg l^-1^ leupeptin and 2 mg l^-1^ sodium orthovanadate). After lysis, samples were centrifuged at 15,000 g for 20 min. Supernatant proteins (15 mg) from total brain regions extracts were loaded in SDS–PAGE and transferred to nitrocellulose membranes (GE Healthcare, LC, UK). Membranes were blocked in TBS-T (150mM NaCl, 20mM Tris-HCl, pH 7.5, 0.5 ml l^-1^ Tween 20) with 50 g l^-1^ phospho-Blocker (Cell Biolabs, San Diego, CA) or 50 g l^-1^ non-fat dry milk and 5 g l^-1^ BSA. Immunoblots were probed with the following antibodies (all diluted 1:1,000 with some exceptions): Rabbit polyclonal antibodies: Pyk2 (074M4755, Sigma), phosphoY402-Pyk2 (44-618G, Invitrogen), PSD-95 (QA210648, Thermo Fisher), and phosphoY1246-GluN2A (4206S, Cell Signaling Technology), phospho-Y1472-GluN2B (0424201-0009761, Cayman antibodies), phosphoY1325-GluN2A (GR14161032, Abcam), GluN2B (2697434, Millipore), GluN2A (NRG1815904, Millipore), phosphoS896-GluN1 (23591, Upstate). Mouse monoclonal antibodies: GluN1 (225310, Millipore). All blots were incubated with the primary antibody overnight at 4ºC by shaking in PBS with 0.2 g l^-1^ sodium azide. After several washes in TBS-T, blots were incubated with secondary anti-rabbit or anti-mouse IgG IRdye800CWcoupled or anti-mouse IgG IRdye700DX-coupled antibodies (1:2,000, Rockland Immunochemicals, USA). Secondary antibody binding was detected by Odyssey infrared imaging apparatus (Li-Cor Inc., Lincoln, NE). For loading control a mouse monoclonal antibody for a-tubulin was used (#083M4847V, 1:20,000; Sigma).

*Statistical analysis.*

Statistical analyses were carried out using the GraphPad Prism 6.0 software. Sample sizes were chosen using a power analysis: 0.05 alpha value, 1 estimated sigma value and 75%. No methods of randomization were used to allocate animals to experimental groups. All experiments were blind-coded to the experimenter. Two-tailed Student’s t-test (95% confidence), one-way ANOVA or two-way ANOVA, with Tukeys's or Bonferroni's post hoc multiple comparison test were performed as required. The post hoc analyses were carried out after verifying normal distribution of the samples for each group by using the Kolmogorov-Smirnov normality test and confirming no differences between variances by using the F test of equality of variances. Two by two comparisons were two-tailed. A *p* value <0.05 was considered significant. All the experiments were replicated two times except for the behavioral experiments that were performed only once.

**References**

1. Giralt A, Coura R,Girault JA. Pyk2 is essential for astrocytes mobility following brain lesion. *Glia* 2016; **64:** 620-634 .

2. Giralt A *et al.* Pyk2 modulates hippocampal excitatory synapses and contributes to cognitive deficits in a Huntington's disease model. *Nat Commun* 2017; **8:** 15592 .

3. Engmann O *et al.* DARPP-32 interaction with adducin may mediate rapid environmental effects on striatal neurons. *Nat Commun* 2015; **6:** 10099 .

**Supplementary figures**





**Supplementary figure 1.** Experimental design graph. An experimental design graph is depicted stating the order of all the procedures performed in mice *in vivo* from figure 1 (**a**) and figure 2 (**b**).

**
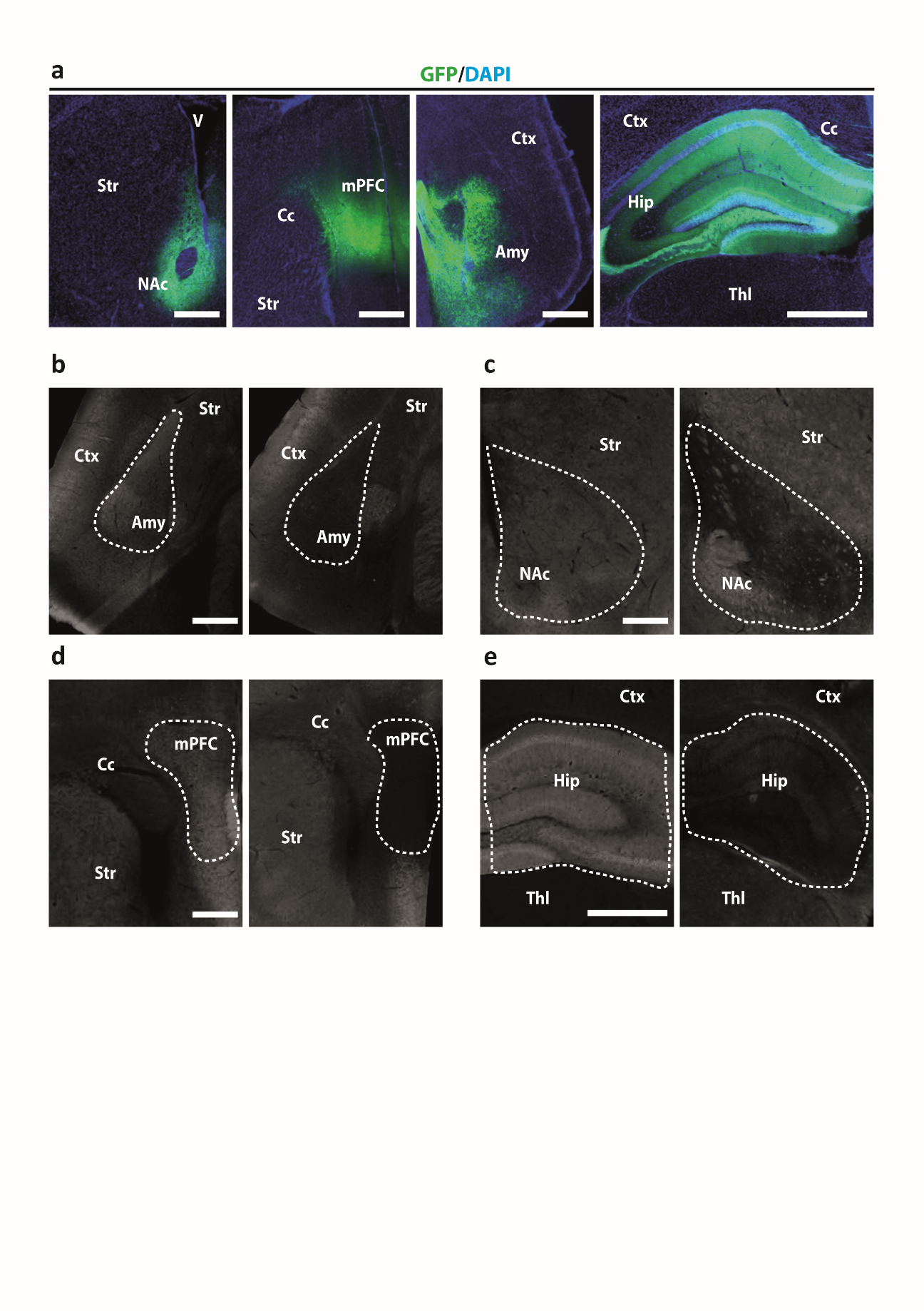
****Supplementary figure 2.** Inactivation of Pyk2 in specific forebrain regions. AAV expressing GFP (AAV-GFP) or GFP-Cre (AAV-Cre) was bilaterally injected in the hippocampus (Hip), nucleus accumbens (NAc), amygdala (Amy) or medial prefrontal cortex (mPFC) of Pyk2^f/f^ mice (**a**) 21 days after the injection, GFP fluorescence (green) was detected in NAc (**a, 1^st^ panel**), mPFC (**a, 2^nd^ panel**), Amy (**a, 3^rd^ panel**), and Hip (**a, 4^th^ panel**). (**b-e**) Pyk2 immunoreactivity in Amy (**b**), NAc (**c**), mPFC (**d**) and Hip (**e**). For each, the left panel shows an AAV-GFP injected brain and the right panel an AAV-Cre-injected brain. Pyk2 immunoreactivity was not altered by AAV-GFP expression, whereas it was decreased by AAV-Cre. All images were obtained with a confocal microscope. Scale bar, 200 µm. Str, striatum; V, lateral ventricle; Cc, corpus callosum; Ctx, cerebral cortex; Thl: Thalamus.
